# House Dust Concentrations of Organophosphate Flame Retardants in Relation to Hormone Levels and Semen Quality Parameters

**DOI:** 10.1289/ehp.0901332

**Published:** 2009-11-13

**Authors:** John D. Meeker, Heather M. Stapleton

**Affiliations:** 1 Department of Environmental Health Sciences, University of Michigan School of Public Health, Ann Arbor, Michigan, USA; 2 Nicholas School of the Environment, Duke University, Durham, North Carolina, USA

**Keywords:** endocrine, epidemiology, exposure, sperm, TDCPP, triphenyl phosphate

## Abstract

**Background:**

Organophosphate (OP) compounds, such as tris(1,3-dichloro-2-propyl) phosphate (TDCPP) and triphenyl phosphate (TPP), are commonly used as additive flame retardants and plasticizers in a wide range of materials. Although widespread human exposure to OP flame retardants is likely, there is a lack of human and animal data on potential health effects.

**Objective:**

We explored relationships of TDCPP and TPP concentrations in house dust with hormone levels and semen quality parameters.

**Methods:**

We analyzed house dust from 50 men recruited through a U.S. infertility clinic for TDCPP and TPP. Relationships with reproductive and thyroid hormone levels, as well as semen quality parameters, were assessed using crude and multivariable linear regression.

**Results:**

TDCPP and TPP were detected in 96% and 98% of samples, respectively, with widely varying concentrations up to 1.8 mg/g. In models adjusted for age and body mass index, an interquartile range (IQR) increase in TDCPP was associated with a 3% [95% confidence interval (CI), −5% to −1%) decline in free thyroxine and a 17% (95% CI, 4–32%) increase in prolactin. There was a suggestive inverse association between TDCPP and free androgen index that became less evident in adjusted models. In the adjusted models, an IQR increase in TPP was associated with a 10% (95% CI, 2–19%) increase in prolactin and a 19% (95% CI, −30% to −5%) decrease in sperm concentration.

**Conclusion:**

OP flame retardants may be associated with altered hormone levels and decreased semen quality in men. More research on sources and levels of human exposure to OP flame retardants and associated health outcomes are needed.

Several chemicals commonly encountered in the environment have been associated with altered endocrine function in animals and humans, and exposure to some endocrine-disrupting chemicals may result in adverse effects on reproduction, fetal/child development, metabolism, neurologic function, and other vital processes (Diamanti-Kandarakis et al. 2009). Recent attention to the potential risks that environmental chemicals may pose to reproductive and developmental health has also been driven by reports of temporal downward trends in semen quality (Carlsen et al. 1992; Swan et al. 2000) and male testosterone levels (Andersson et al. 2007; Travison et al. 2007); increased rates of development anomalies of the reproductive tract, specifically hypospadias and cryptorchidism (Paulozzi 1999); and increased rates of testicular cancer (Adami et al. 1994; Bergström et al. 1996; Huyghe et al. 2003). Public and scientific concern also stems from recent reports of inexplicable increases in the rates of thyroid cancer (Davies and Welch 2006; Enewold et al. 2009), congenital hypothyroidism (Harris and Pass 2007), and neurologic development disorders such as autism (Hertz-Picciotto and Delwiche 2009). Not only do these studies report temporal trends, but many also describe wide geographic variability in these measures and trends, which provides further evidence that environmental factors may play a role.

Flame retardants are used in construction materials, furniture, plastics, electronics equipment, textiles, and other materials. Until recently, polybrominated diphenyl ethers (PBDEs) accounted for a large proportion of flame retardants used in polyurethane foam and electronic applications [Consumer Product Safety Commission (CPSC) 2006]. However, in the past several years, common PBDE mixtures (i.e., pentaBDE and octaBDE) have been banned or voluntarily phased out in the United States and many parts of the world because of their persistence, bioaccumulation, and evidence for adverse health effects including endocrine disruption and altered fetal development (Meeker et al. 2009a, 2009b). Thus, the use of alternate flame retardants has been on the rise (Stapleton et al. 2008), as has scrutiny related to the potential environmental and human health consequences of alternate flame retardants. Compared with PBDEs and other brominated flame retardants (e.g., hexabromocyclododecane and tetrabromobisphenol A), organophosphorus (OP) flame retardants have received little attention with regard to human exposure and potential health effects. Trichloroalkyl phosphates, such as tris(1,3-dichloro-2-propyl) phosphate (TDCPP), and triaryl phosphates, such as triphenyl phosphate (TPP), continue to be used as flame retardants and plasticizers in a wide variety of applications resulting in widespread environmental dispersion (Reemtsma et al. 2008). Production and use of OP flame retardants has surpassed that of PBDEs in Europe (Reemtsma et al. 2008), and annual production of both TDCPP and TPP in the United States has been estimated to be between 10 and 50 million pounds per year [U.S. Environmental Protection Agency (EPA) 2006]. As with PBDEs, OPs such as TDCPP and TPP are used as additive flame retardants that can be released into the surrounding environment over time (U.S. EPA 2005). Recent studies have reported that concentrations of OP flame retardants measured in house dust are on the same order of magnitude as PBDEs, and for some OPs, such as TPP, concentrations greatly exceed those of PBDEs (Reemtsma et al. 2008; Stapleton et al. 2009). Given that house dust is a primary source of exposure to PBDEs (Johnson-Restrepo and Kannan 2009; Lorber 2008) and that PBDEs can be detected in the blood of nearly all individuals in the general population (Sjodin et al. 2008), human exposures to OP flame retardants are also likely to be widespread.

The presence of TDCPP was reported in a significant proportion of human seminal plasma samples nearly three decades ago (Hudec et al. 1981), and high doses of OP flame retardants have been associated with adverse reproductive, neurologic, and other systemic effects (e.g., altered thyroid and liver weights) in laboratory animals [CPSC 2006; National Research Council (NRC) 2000; U.S. EPA 2005]. To our knowledge, no studies exploring associations between nonoccupational exposure to OP flame retardants and human health end points have been conducted to date, although evidence exists for endocrine and reproductive effects in relation to other OP compounds. In the present study, we measured two OP flame retardants (TDCPP and TPP) in house dust and assessed relationships with hormone levels and semen quality parameters among men recruited from an infertility clinic as part of an ongoing study of environmental influences on reproductive health.

## Methods

### Subject recruitment

Our study was conducted on a subset of men participating in an ongoing study of environmental factors in reproductive health. Details of subject recruitment have been described previously (Meeker et al. 2007). Briefly, men between 18 and 54 years of age were recruited from the Vincent Memorial Andrology laboratory at Massachusetts General Hospital and invited to participate in the study. Applicable requirements involving human subjects were followed. Institutional review board approval was obtained from each participating institution, and all subjects signed an informed consent. Participants included men from couples who were infertile due to a male factor, a female factor, or a combination of both. Approximately 65% of eligible men agreed to participate. Exclusionary criteria included prior vasectomy or current use of exogenous hormones.

### Dust sample collection and analysis

The use of vacuum bags from the homes of study participants is a validated and cost-effective method of collecting household dust samples for a range of organic and inorganic chemicals in epidemiologic studies (Colt et al. 1998, 2008). We collected used household vacuum bags from men participating in the parent study between 2002 and 2007; existing vacuum bags were collected in the home by participants upon enrollment in the study. We analyzed vacuum bags from 50 participants, selected independently of fertility status or projected exposure levels, for OP flame retardants.

Internal and surrogate standards used in this study were purchased from Wellington Laboratories (Guelph, Ontario, Canada), Cambridge Isotope Laboratories (Andover, MA), and Chiron (Trondheim, Norway). TDCPP was purchased from Chem Service (West Chester, PA), and TPP (99% pure) was purchased from Sigma-Aldrich (St. Louis, MO). All solvents used throughout this study were HPLC (high-performance liquid chromatography) grade. Approximately 0.3–0.5 g sieved (150–μm screen) dust was accurately weighed, spiked with 50–100 ng of two internal standards [4′fluoro-2,3′,4,6-tetrabromodiphenyl ether (F-BDE 69) and ^13^C-labeled decabromodiphenyl ether (^13^C-BDE 209)], and extracted in stainless steel cells using pressurized fluid extraction (ASE 300, Dionex Inc., Sunnyvale, CA). Cells were extracted three times with 50:50 dichloromethane:hexane at a temperature of 100°C and at 1,500 psi. Final extracts were reduced in volume to approximately 1.0 mL using an automated nitrogen evaporation system (Turbo Vap II; Caliper Life Sciences, Hopkinton, MA). Dust extracts were purified by elution through a glass column containing 4.0 g 6% deactivated alumina. All analytes were eluted with 50 mL of a 50:50 mixture of dichloromethane:hexane. The final extract was then reduced in volume to 0.5 mL, and 50 ng of the recovery standard, 2,2′,3,4,5,5′-hexachloro[13C12]diphenyl ether (^13^C-CDE 141), was added prior to gas chromatography-mass spectroscopy (GC/MS) analysis.

We analyzed samples using GC/MS operated in either electron impact mode for TPP, or electron capture negative ionization mode for TDCPP. A 0.25-mm (i.d.) × 15 m fused silica capillary column coated with 5% phenyl methylpolysiloxane (0.25 μm film thickness) was used for separation of the analytes. Pressurized temperature vaporization injection was employed in the GC. The inlet was set to a temperature of 80°C for 0.3 min; a 600°C/min ramp to 275°C was then employed to efficiently transfer the samples to the head of the GC column. The oven temperature program was held at 40°C for 1 min followed by a temperature ramp of 18°C/min to 250°C, followed by a temperature ramp of 1.5°C/min to a temperature of 260°C, followed by a final temperature ramp of 25°C/min to 300°C, which was held for an additional 20 min. The transfer line temperature was maintained at 300°C, and the ion source was held at 200°C. ^13^C-BDE 209 was monitored by *m/z* 494.6 and 496.6; TDCPP was quantified by monitoring *m/z* 319 and 317, and TPP was quantified by monitoring *m/z* 326 and 325. As further confirmation, all ion ratios were monitored and were within 20% of their expected values compared with authentic standards.

As part of our quality assurance criteria, we examined levels of these specific analytes in laboratory blanks (*n* = 4), replicate samples (*n* = 3), and in matrix spikes (*n* = 3). Sample measurements were blank-corrected by subtracting the average level measured in the laboratory blanks. Blank levels for TDCPP and TPP were 11.7 ± 6.6 and 15.7 ± 11.9 ng, respectively. Method detection limits were calculated as three times the standard deviation of the blank levels. Matrix spikes were prepared by adding between 25 and 100 ng TDCPP and TPP to ASE cells filled with sodium sulfate powder. Matrix spikes were extracted using the same method used for dust and examined for percent recovery using 50 ng ^13^C-CDE 141 as an internal standard. Recoveries averaged 86 ± 7 and 89 ± 2% for TDCPP and TPP, respectively.

### Serum hormones

One nonfasting blood sample was drawn and centrifuged, and the serum was stored at −80°C until analysis. The hormone analytical methods have been described previously (Meeker et al. 2007). Briefly, we measured testosterone directly using the Coat-A-Count radioimmunoassay kit (Diagnostics Products, Los Angeles, CA); sex hormone binding globulin (SHBG) using a fully automated chemiluminescent immunometric assay (Immulite; DPC, Inc., Los Angeles, CA); and inhibin B using a commercially available, double antibody, enzyme-linked immunosorbent assay (Oxford Bioinnovation, Oxford, UK). Serum luteinizing hormone (LH), follicle-stimulating hormone (FSH), estradiol, prolactin, free thyroxine (T_4_), total triiodothyronine (T_3_), and thyrotropin (TSH) concentrations were determined by microparticle enzyme immunoassay using an automated Abbott AxSYM system (Abbott Laboratories, Chicago, IL). The free androgen index (FAI) was calculated as the molar ratio of total testosterone to SHBG.

### Semen quality

Semen samples were analyzed for sperm concentration and motion parameters by a computer-aided semen analyzer (HTM-IVOS, version 10HTM-IVOS; Hamilton-Thorne Research, Beverly, MA). Setting parameters and the definition of measured sperm motion parameters for the computer-aided semen analyzer were established by manufacturer. To measure both sperm concentration and motility, 5 μL of semen from each sample was placed into a prewarmed (37°C) Makler counting chamber (Sefi Medical Instruments, Haifa, Israel). We analyzed a minimum of 200 sperm cells from at least four different fields from each specimen. Motile sperm was defined as World Health Organization (WHO) grade a sperm (rapidly progressive with a velocity ≥ 25 μm/sec at 37°C) and grade b sperm (slow/ sluggish progressive with a velocity ≥ 5 μm/sec but < 25 μm/sec) (WHO 1999). For sperm morphology, at least two slides were made for each fresh semen sample. The resulting thin smear was allowed to air dry for 1 hr before staining with a Diff-Quik staining kit (Dade Behring AG, Dudingen, Switzerland). We performed morphologic assessment with a Nikon microscope using an oil immersion 100× objective (Nikon Company, Tokyo, Japan). We counted a minimum of 200 sperm cells from two slides for each specimen.

### Statistical analysis

We analyzed data using SAS, version 9.1 (SAS Institute Inc., Cary, NC). In preliminary analyses, Pearson correlation coefficients were calculated to assess bivariate relationships between OP concentrations in house dust, serum hormone levels, and semen quality parameters. Spearman rank correlation coefficients were also calculated and were compared with the Pearson correlations for consistency. Spearman correlations are presented only in instances where they were inconsistent with the Pearson correlation. Multivariable linear regression was then used to assess these relationships while adjusting for potential confounding variables. Serum levels of several hormones (testosterone, inhibin B, estradiol, free T_4_, and total T_3_), sperm motility, and sperm morphology closely approximated normality and were used in statistical models untransformed, whereas the distributions of several other hormones (FSH, LH, SHBG, FAI, prolactin, and TSH) and sperm concentration were skewed right and transformed to the natural log (ln) for statistical analyses. OP concentrations in dust were also ln-transformed. Dust samples with nondetectable OP concentrations were assigned a value equal to one-half the limit of detection (LOD). All multivariable models were adjusted for age and body mass index (BMI) as continuous variables. The models for semen quality parameters were also adjusted for abstinence period leading up to the collection of the semen sample, entered as a 5-level ordinal variable. Current smoking, time of day, season in which blood samples were collected from participants, and alcohol intake (number of drinks per week) were also considered but did not act as confounders and were not retained in the final models. To improve interpretability, the regression coefficients were exponentiated and expressed as a percent change in the dependent variable (i.e., change in hormone levels or semen quality parameter relative to the study population median) for an interquartile range (IQR) increase in dust OP concentration.

## Results

Of the 50 dust samples collected, we detected TDCPP and TPP in 48 (96%) and 49 (98%) samples, respectively. The distribution of measured TDCPP and TPP concentrations are presented in Table 1. Concentrations of TDCPP and TPP were positively (right)-skewed and varied widely, from < LOD (107 and 173 ng/g, respectively) to maximum values of 56 μg/g and 1,800 μg/g, respectively. The distribution of age, BMI, serum hormone levels, and semen quality parameters among the men in the study are also presented in Table 1.

**Figure 1:**
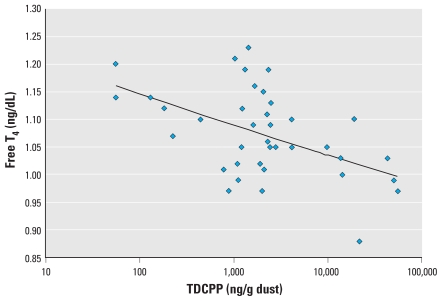
Scatterplot of TDCPP in house dust and serum free T_4_ (*n* = 38; *r* = −0.50; *p* = 0.002). The inverse relationship remained when one outlying free T_4_ value (0.88 ng/dL) was excluded from the analysis (*r* = −0.45, *p* = 0.005).

**Figure 2:**
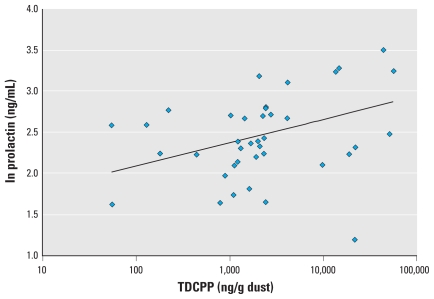
Scatterplot of TDCPP in house dust and serum prolactin (*n* = 38; *r* = 0.43; *p* = 0.0007).

**Figure 3:**
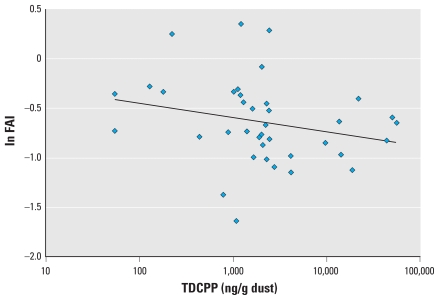
Scatterplot of TDCPP in house dust and serum FAI (*n* = 38; *r* = 0.24; *p* = 0.14).

**Figure 4:**
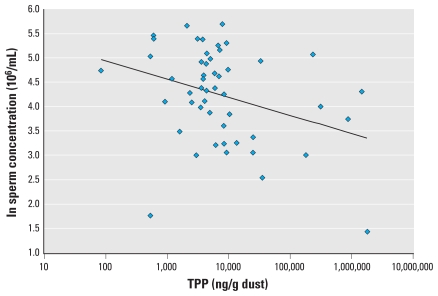
Scatterplot of TPP in house dust and sperm concentration (*n* = 50, *r* = 0.33, *p* = 0.02). The inverse association remained (*r* = −0.31, *p* = 0.03) when three men with sperm concentration of < 20 million/mL were excluded from the analysis.

Semen quality parameters were available for all 50 men with OP dust measures, and hormone levels were available for 38 of the men. In preliminary correlation analysis, TDCPP and TPP were moderately correlated (Pearson *r* = 0.56, *p* = 0.0001; Spearman *r* = 0.33, *p* = 0.02). TDCPP was inversely associated with free T_4_ (Figure 1; correlation coefficients in the figures are Pearson correlations) and positively associated with prolactin (Figure 2). We also found a suggestive inverse association between TDCPP and FAI, as shown in Figure 3, although the Spearman rank correlation for this relationship was stronger than the Pearson correlation (Spearman *r* = −0.33, *p* = 0.04). TPP was positively correlated with prolactin (not shown; Pearson *r* = 0.37; *p* = 0.02) and inversely associated with sperm concentration (Figure 4). The inverse association remained when the three men with a sperm concentration below the commonly used reference criteria of 20 million sperm/mL (WHO 1999) were removed from the analysis (Pearson *r* = −0.31, *p* = 0.03).

In multivariable linear regression models adjusted for age and BMI, the inverse association between TDCPP and free T_4_ and the positive association between TDCPP and prolactin remained (Table 2). An IQR increase in dust TDCPP concentration was associated with a 2.8% [95% confidence interval (CI), −4.6% to −1.0%] decline in free T_4_ relative to the population median. This relationship was robust to the exclusion of an outlying free T_4_ value (0.88 ng/dL) from the model (not shown; *p* = 0.01). An IQR increase in TDCPP was also associated with a significant 17.3% (95% CI, 4.1–32.2%) increase in serum prolactin. The suggestive inverse association between TDCPP and FAI was no longer evident in the multivariate models (Table 2). The inconsistency between Pearson and Spearman correlations in the preliminary analysis led us to assess the potential for influential values in the results of parametric models. When excluding three subjects that had studentized residuals of > 2 or less than −2 in the original model, an IQR increase in TDCPP was associated with a suggestive 6.3% decline in FAI (95% CI, −13.8 to 1.9%; *p* = 0.13).

The inverse association between TPP and sperm concentration was not sensitive to the adjustment for covariates (Table 3). When adjusting for age, BMI, and abstinence period, an IQR increase in TPP was associated with an 18.8% (95% CI, −30.1 to −4.5%) decline in sperm concentration. This inverse relationship remained when excluding three men with sperm concentration below the reference level of 20 million/mL (not shown; *p* = 0.02).

## Discussion

In the present study, we found that OP flame retardants were detected in nearly 100% of house dust samples collected from 50 homes, and OP concentrations varied widely between homes. We recently demonstrated that, in these samples, geometric mean TDCPP and TPP concentrations were on the same order of magnitude as the sum of 34 PBDE congeners (Stapleton et al. 2009). TPP concentrations were considerably higher than PBDE concentrations and ranged up to maximum concentration of 1.8 mg/g compared with 0.04 mg/g for sum of PBDEs. Because of the ubiquity of these compounds in homes and other microenvironments (Marklund et al. 2003, 2005; Takigami et al. 2009), their toxicity potential should be considered more fully, especially as replacements for recently banned or withdrawn PBDE formulations are sought.

We found an inverse association between TDCPP concentrations in house dust and serum free T_4_ levels. Thyroid hormones are vital to a number of physiologic processes including metabolism, reproduction, cardiovascular health, and neurodevelopment. Because thyroid hormone insufficiency can have serious adverse effects on a number of vital physiologic functions, even chemicals that cause only a subtle shift in thyroid hormone levels should be considered carefully in terms of societal impact at the population level (Miller et al. 2009). We also found positive relationships between both TDCPP and TPP with prolactin. Prolactin is a protein hormone that serves a number of important functions involving reproduction, metabolism, maintenance of homeostasis in immune responses, osmotic balance, and angiogenesis (Ben-Jonathan et al. 2008; Freeman et al. 2000) and is increasingly becoming used as a measure of neuroendocrine/dopaminergic function in environmental and occupational epidemiology studies. (de Burbure et al. 2006; Meeker et al. 2009c). Because dopamine is responsible for inhibiting prolactin secretion, increased circulating levels of prolactin may reflect deficiencies in dopamine release, transport, or uptake (Felt et al. 2006). Finally, in these data we also observed that an IQR increase in house dust TPP was associated with a substantial (19%) decline in sperm concentration. These findings may have substantial public health implications, given the likelihood of exposure to TPP among the general population, but more human and animal studies are needed to confirm our results.

Toxicology data relevant to the end points explored in the present study are limited (CPSC 2006; NRC 2000; U.S. EPA 2005). TPP binds to the androgen receptor with moderate affinity (Fang et al. 2003) and activates enzymes involved in steroid hormone metabolism *in vitro* (Honkakoski et al. 2004). In addition, in rat studies butylated TPP has been associated with endocrine effects, including reduced male fertility and altered female reproductive cycles (Latendresse et al. 1994). Rats dosed with high levels of TDCPP had altered thyroid weights and an increased thyroid/body weight ratio compared with control animals, and high-dose males demonstrated a significant increase in testicular interstitial-cell tumors and had increased histopathologic abnormalities in the testis, epididymis, and seminal vesicle (NRC 2000).

We can also compare our findings with effects reported in association with other OP compounds. Consistent with our observation of an inverse association between TPP and sperm concentration, other OP flame retardants and plasticizers such as tricresyl phosphate are reproductive toxicants that have caused marked reductions in male fertility and semen quality measures in laboratory animals (NRC 2000). Our findings are also consistent with several reports on the more well-studied OP insecticides. For example, in our previous work among a larger and overlapping group of men from the ongoing study, urinary levels of 3,5,6-trichloro-2-pyridinol, a urinary metabolite of the OP insecticides chlorpyrifos and chlorpyrifos-methyl, were associated with increased prolactin (Meeker et al. 2008) and reduced free T_4_ (Meeker et al. 2006a), FAI (Meeker et al. 2006b), and sperm motility (Meeker et al. 2004). A variety of OP pesticides have been linked to decreased semen quality in studies of occupationally exposed men (Padungtod et al. 2000; Recio-Vega et al. 2008; Yucra et al. 2006, 2008). Case studies of patients with acute OP pesticide poisoning have also reported decreased levels of circulating thyroid hormones and increased prolactin (Guven et al. 1999; Satar et al. 2005). This is consistent with animal studies that reported inverse associations between chlorpyrifos and T_4_ levels in mice and sheep (De Angelis et al. 2009; Rawlings et al. 1998), and studies that reported increased prolactin levels, decreased dopamine and testosterone levels, and damaged male reproductive organs in rats exposed to the OP insecticide quinalphos (Sarkar et al. 2000). Thus, the relationships observed in the present study may be consistent with those reported for OP insecticides. However, compared with OP triesters used as insecticides, OP flame retardants are more stable against hydrolysis, which makes them more persistent in the environment (Reemtsma et al. 2008). This potential for environmental persistence, combined with the widespread use and distribution of these compounds and the potential for adverse reproductive and neuroendocrine effects, serves as a further indication that more research is needed on the potential health effects resulting from exposure to OP flame retardants.

Our study has a number of limitations. First, the sample size was relatively small, which may have limited our ability to detect subtle associations between OP levels and hormone or semen quality markers. Second, because this represents the first study to assess the relationship between OP flame retardants and endocrine/reproductive outcomes in humans, a number of comparisons were made. Thus, the observation of a statistical relationship due to chance cannot be ruled out. Third, the study was cross-sectional in nature, so we cannot make conclusions regarding the temporality of the relationships observed. Fourth, we cannot rule out the presence of unmeasured confounders or coexposures that may explain our reported findings. However, we considered a number of potential confounding factors in the multivariable models. In addition, we previously reported that TDCPP and TPP were only moderately correlated with PBDE congeners and other brominated flame retardants (all correlation coefficients were ≤ 0.4) (Stapleton et al. 2009). Finally, we used house dust concentrations to estimate exposure to TDCPP and TPP. Because data on the prevalence, sources, and pathways of human exposure to TDCPP and TPP are lacking, this approach may be associated with exposure measurement error. However, if the measurement error were nondifferential, it would be expected to dilute exposure–outcome relationships toward the null (Armstrong 2004). Also, TDCPP and TPP are used (in a manner similar to that of PBDEs) as additive flame retardants in furniture foams, textiles, plastics, and electronics, which may result in exposure sources and pathways similar to those of PBDEs in the home and in other microenvironments where house dust plays a primary role in aggregate exposure (Johnson-Restrepo and Kannan 2009; Lorber 2008). Sensitive and specific biomarkers of exposure to these OP flame retardants are needed, as are studies comparing the contribution of OP concentrations in house dust and other environmental media (e.g., air, diet) to biomarker levels to determine important pathways of exposure and the most relevant media and time windows for the estimation of exposure in epidemiologic studies.

## Conclusion

We found evidence that concentrations of OP flame retardants in house dust may be associated with altered hormone levels and decreased sperm concentration. More research is needed to determine the extent and sources of human exposure to OP flame retardants and associated effects on human health.

## Figures and Tables

**Table 1:** Distribution of TDCPP and TPP in house dust, and participant age, BMI, hormone levels, and semen quality parameters.

			Selected percentiles						
Variable	*n*	Mean	Minimum	10th	25th	50th	75th	90th	Maximum
OP									
TDCPP (ng/g dust)	50	1,880^a^	< 107^b^	202	937	1,752	2,959	20,415	56,090
TPP (ng/g dust)	50	7,400^a^	< 173^b^	760	3,100	5,470	9,830	208,200	1,798,100
Age (years)	50	36.5	28	31	33	37	40	42	46
BMI (kg/m^2^)	50	26.8	20.3	21.6	23.0	25.9	29.7	33.0	42.4
Hormone									
Free T_4_ (ng/dL)	38	1.07	0.88	0.97	1.02	1.07	1.13	1.19	1.23
Total T_3_ (ng/mL)	38	0.92	0.74	0.76	0.80	0.93	1.02	1.10	1.23
TSH (μIU/mL)	38	1.43^a^	0.45	0.64	1.04	1.34	1.91	3.07	4.95
FSH (IU/L)	38	9.1^a^	3.1	4.3	6.5	8.8	13.1	18.0	29.5
LH (IU/L)	38	9.2^a^	3.7	5.2	6.6	9.7	11.7	16.1	20.4
Inhibin B (pg/mL)	38	209	67.6	115	142	177	241	308	702
Testosterone (ng/dL)	38	391	148	235	325	402	470	515	633
SHBG (nmol/mL)	38	24.4^a^	7.4	9.7	18.9	26.0	35.9	44.0	60.5
FAI	38	53.0^a^	19.5	32.6	42.1	49.9	69.4	92.9	143
Estradiol (pg/mL)	38	20.5	< 20.0^b^	< 20.0^b^	< 20.0^b^	21.5	28.0	38.0	48.0
Prolactin (ng/mL)	38	11.8^a^	5.1	5.7	9.2	11.0	15.2	25.9	33.6
Sperm concentration (10^6^/mL)	50	70.5^a^	4.3	21.2	37.6	78.8	148	219	301
Sperm motility (% motile)	50	49.7	12	22	38	51	64	76	81
Sperm morphology (% normal)	50	6.9	0	2	4	7	10	14	17

a Geometric mean.

b LOD for TDCPP = 107 ng/g, for TPP = 173 ng/g, and for estradiol = 20 pg/mL.

**Table 2:** Adjusted^a^ regression coefficients (95% CIs) for percent change in hormone level (relative to population median) associated with an IQR increase in dust TDCPP or TPP concentration (*n* = 38).

	TDCPP^b^	*p*-Value	TPP^b^	*p*-Value
Free T_4_	−2.8 (−4.6 to −1.0)	0.004	−1.1 (−2.4 to 0.2)	0.11
Total T_3_	3.3 (−0.4 to 6.9)	0.08	2.0 (−0.5 to 4.5)	0.12
TSH^b^	3.1 (−11.8 to 20.5)	0.70	0.5 (−9.6 to 11.6)	0.93
FSH^b^	8.3 (−5.2 to 25.4)	0.23	4.7 (−4.5 to 14.9)	0.34
LH^b^	5.5 (−6.4 to 18.9)	0.36	3.5 (−3.4 to 12.2)	0.31
Inhibin B	−7.0 (−25.7 to 11.7)	0.45	−4.6 (−17.1 to 8.0)	0.46
Testosterone^c^	−2.1 (−8.5 to 4.3)	0.50	1.3 (−3.1 to 5.6)	0.55
Estradiol	8.7 (−5.3 to 22.7)	0.21	6.7 (−2.6 to 16.1)	0.15
SHBG^b^	2.7 (−8.9 to 17.3)	0.61	2.3 (−5.6 to 10.9)	0.53
FAI^b^	−5.2 (−15.9 to 5.5)	0.31	−1.2 (−8.8 to 5.9)	0.71
Prolactin^b^	17.3 (4.1 to 32.2)	0.008	9.7 (2.3 to 18.9)	0.02

a Adjusted for age and BMI.

b Variable ln-transformed in statistical analysis.

c Models for testosterone also adjusted for ln-transformed SHBG.

**Table 3:** Adjusted^a^ regression coefficients (95% CIs) for percent change in semen quality parameter (relative to population median) associated with an IQR increase in dust TDCPP or TPP concentration (*n* = 50).

	TDCPP^b^	*p*-Value	TPP^b^	*p*-Value
Sperm concentration^b^	−13.6 (−32.0 to 8.3)	0.19	−18.8 (−30.1 to −4.5)	0.01
Sperm motility	−4.9 (−14.0 to 4.4)	0.29	−1.4 (−7.8 to 5.1)	0.67
Sperm morphology	−4.2 (−19.6 to 11.4)	0.59	−5.8 (−16.5 to 5.0)	0.28

a Adjusted for age, BMI, and abstinence period.

b Variable ln-transformed in statistical analysis.
